# Biodistribution of therapeutic extracellular vesicles

**DOI:** 10.20517/evcna.2023.12

**Published:** 2023-04-19

**Authors:** Dhanu Gupta, Oscar P.B Wiklander, Matthew J.A Wood, Samir El-Andaloussi

**Affiliations:** ^1^Department of Paediatrics. University of Oxford, Oxford OX3 7TY, UK.; ^2^Biomolecular Medicine, Division of Biomolecular and Cellular Medicine, Department of Laboratory Medicine, Karolinska Institutet, Huddinge 14151, Sweden.

**Keywords:** Exosomes, *in vivo* biodistribution, extracellular vesicles, targeted delivery, CNS targeting

## Abstract

The field of extracellular vesicles (EVs) has seen a tremendous paradigm shift in the past two decades, from being regarded as cellular waste bags to being considered essential mediators in intercellular communication. Their unique ability to transfer macromolecules across cells and biological barriers has made them a rising star in drug delivery. Mounting evidence suggests that EVs can be explored as efficient drug delivery vehicles for a range of therapeutic macromolecules. In contrast to many synthetic delivery systems, these vesicles appear exceptionally well tolerated *in vivo*. This tremendous development in the therapeutic application of EVs has been made through technological advancement in labelling and understanding the *in vivo* biodistribution of EVs. Here in this review, we have summarised the recent findings in EV *in vivo* pharmacokinetics and discussed various biological barriers that need to be surpassed to achieve tissue-specific delivery.

## INTRODUCTION

Extracellular vesicles (EVs) are a heterogeneous group of membranous nanoparticles actively secreted by all cells. Based on their biogenesis, EVs are differentiated into three main subtypes: microvesicles (MVs), exosomes, and apoptotic bodies^[1,2]^. In addition to this classification, it is becoming increasingly evident that various sub-populations of vesicles exist, especially different types of exosomes^[3]^. EVs contain biologically active molecules, including various RNAs, proteins and bioactive lipids derived from their producer cell^[4]^. The intercellular transfer of these functional cargo renders EVs an essential player in physiology. As nature’s very own nanoparticles, EVs intrinsically benefit from stability in circulation, biocompatibility, immune tolerance and the ability to cross biological barriers to reach hard-to-target organs such as the brain and muscles^[5,6]^. These unique properties of EVs have inspired many to use them as a next-generation drug delivery tool. Therapeutic EV research has seen tremendous development in the past decade, from *in vitro* studies towards pre-clinical models to clinical trials^[5]^. Even so, the road towards successful clinical translation has various obstacles, primarily due to the knowledge gap in EV biology. One of the primary challenges is to achieve reliable detection of exogenous EVs *in vivo* and to understand their *in vivo* pharmacokinetic properties. This is particularly challenging given the fact that the extracellular space is rich in various nanoparticulate species ranging from apoptotic bodies to exosomes, lipoprotein complexes, and ribonucleoprotein complexes, *etc*.^[7]^. Therefore, tools for characterizing their spatiotemporal properties have been developed to dissect their biological input in pathophysiology, development, and biotherapeutic delivery^[7]^. By harnessing the power of these tools, various studies have shed light on EV *in vivo* biodistribution and underpinned various factors governing *in vivo* biodistribution. This review discusses the current state of the art for the technologies used in purifying, characterizing and labelling EVs, followed by general trends *in vivo* biodistribution of EVs, and discusses various biological barriers EVs experience *in vivo* before entering specific tissue. Furthermore, the review discusses the major challenges that need to be addressed to achieve targeted delivery of EVs.

## PURIFICATION OF EVs

Purification of EVs is a significant challenge as the extracellular environments in both biofluids and cell culture supernatants are rich in protein aggregates, lipoprotein complexes, non-EV bound RNA, cell debris and a heterogeneous pool of EVs^[8-11]^. Each of these components can have a physiological effect on the recipient cell^[12]^. Therefore, it is critical to have a robust EV purification method that enriches EVs to dissect their biological, therapeutic, and diagnostic roles. Different purification methods may need to be combined based on the desired EV application to achieve a highly pure EV preparation^[13-15]^. For example, EV purification from plasma is challenging as it is rich in albumin, lipoproteins and protein aggregates, and these contaminants overlap with EVs with regard to various physiochemical parameters^[16-18]^. Hence, the purification of EVs is of great importance in all areas of EV research. Since the first use of high-speed centrifugation for purifying EVs, the purification methods have evolved rapidly with the help of expanding knowledge about EVs and can now be isolated based on various physicochemical properties such as density, size, charge, or affinity to specific biomolecules on the surface of EVs^[18]^.

### Ultracentrifugation

Centrifugation-based EV purification is still the most widely used method across the EV field^[19]^. The traditional process involves a series of low-speed spins to clear cell culture supernatants or biological fluids from cells, apoptotic cell debris, and other large microscopic particles. The precleared supernatant is further processed using multiple high-speed centrifugations ranging from 10,000 g to 200,000 g to recover large vesicles to small vesicles, including exomeres^[20,21]^. Since the separation of EVs is based on size and density, protein aggregates tend to copurify with EVs^[22]^. For enhanced EV purity, density gradient centrifugations with 30%-60% sucrose cushion or iodixanol can be employed to remove vesicle-free proteins or protein-RNA aggregates^[23,24]^. Although ultracentrifugation (UC) on a density gradient yields pure EV populations, the method itself is variable as the yields are dependent on user handling and experience. Furthermore, particle disruption, aggregation, and lack of scalability are still significant limitations of the ultracentrifugation-based purification methods^[25,26]^.

### Size exclusion chromatography

Size exclusion chromatography (SEC) is a widely used chromatography method that is used across various research fields. SEC serves as a means to separate molecules based on molecular size. Briefly, the sample which acts as a mobile phase is passed through a porous stationary phase^[27]^. Smaller particles will be able to traverse through more pores as compared to bigger particles, hence resulting in differential elution profiles where bigger particles will take a shorter path and elute first, followed by smaller vesicles and then non-exosomal proteins^[26]^. Pore size and density of the stationary phase are based on the polymer used and can be modulated by selecting from the number of gel polymers available such as crosslinked dextran, agarose, or allyldextran^[28]^. Due to the limitation of mobile phase volume, which can be subjected to SEC, a pre-processing step such as ultrafiltration to concentrate the sample is often performed^[26,29,30]^. Usually, precleared cell culture supernatants are subjected to ultrafiltration devices either with dead-end or tangential flow filtration systems with a molecular weight cut-off ranging from 10 to 1,000 kDa. Dead-end filtration is more suitable for small-scale applications, while tangential flow filtration is more appropriate for large-scale production^[31,32]^. Furthermore, using ultrafiltration with higher molecular weight cut-off values, relatively pure and intact vesicle preparations can be obtained, hence further improving the efficiency of the downstream purification system^[33]^. EVs purified by SEC have better integrity and purity as compared to UC-based approaches, as demonstrated by several studies^[25,26]^. In addition, the scalability of SEC makes it a promising candidate for EVs purification for GMP production^[34-36]^. However, since the separation of EVs in SEC is based on size, the risk of co-elution of VLDL, chylomicrons, and LDL is relatively high in the case of EV purification from plasma^[17]^. Furthermore, due to the long processing time and lack of throughput, the applicability of SEC in EV-based diagnostics is complicated.

### Alternative methods for EV purification

Apart from using density and size, alternative methods have emerged which utilize molecular, biophysical or biochemical attributes of EVs to segregate EVs from other non-vesicular contaminants and aim to delineate the heterogeneity of EVs. One way of efficiently capturing EVs in biological samples is to target specific surface markers known for a particular EV population^[18]^. Up until now, a wide range of EV markers has been identified, which has led to the development of various immune affinity-based approaches for EV purification. The most commonly used immunoaffinity approaches are directed towards EV-specific tetraspanins such as CD9, CD81, and CD63^[37]^. In addition, some other protein targets have also been used for immunoaffinity purification of EVs, such as MHC antigen^[38]^ and heat shock proteins^[39]^. Apart from protein-based targets, targeting lipids such as phosphatidylserines by Annexin V^[40]^ or targeting proteoglycans or other glycocalyx structures by heparin^[41]^, and lectins^[42-44]^ have been used for affinity-based EV purification.

An alternative method employing low-speed centrifugation for EV isolation is precipitation, either by adding polyethylene glycol or organic solvent (protein organic solvent precipitation technique)^[45,46]^. However, these approaches precipitate both vesicular and non-vesicular proteins in the sample. Therefore, they are not considered EV purification methods but rather methods used to enrich EVs.

Immunoaffinity-based EV purification allows for efficient isolation of pure EVs from complex biological fluids in a high throughput manner, with minimum user dependency on purity and yield, and has therefore been deemed ideal for EV-based diagnostic applications^[47,48]^. For therapeutic purposes, efforts are being made to increase the scalability and nondestructive retrieval of EVs from the affinity columns^[49,50]^.

In addition to immunoaffinity, several other technologies have been developed or repurposed for EV isolation. Amongst others, these include ion-exchange chromatography^[51,52]^ asymmetric flow field fractionation^[53,54]^, and microfluidic-based systems^[55,56]^.

## EV CHARACTERIZATION

As aforementioned, EVs are a heterogenous pool of vesicles that contain proteins, a variety of nucleic acid species ranging from small RNA to full-length mRNA and DNA, and lipids^[4]^. Since EVs are smaller than the wavelength of visible light, reliable detection of EVs is challenging. However, over the years, various methodologies have been developed to achieve reliable quantification of EVs^[57]^.

Levels of any of the biomolecular cargo or the vesicle as a structure can serve as a basis for EV quantification. Based on this, various means of quantifying EVs have been developed. Technologies which measure hydrodynamic sizes, such as nanoparticle tracking analysis (NTA)^[58]^ and resistive pulse sensing^[59]^, are sensitive and provide a robust means for sizing and determining EV concentrations. However, these methods fail to distinguish an EV from other non-vesicular particles of similar size. Hence the specificity of the assay is entirely dependent on the choice of purification method, as certain methods tend to copurify nonlipoprotein complexes and protein aggregates^[22,60,61]^.

Recently, flow cytometry-based applications have emerged to quantify EVs at a single vesicle level^[62-66]^. However, considering the size of an EV, especially the small EVs, the amount of light scattered fails to trigger the sensor on conventional flow cytometers. Therefore, a flow cytometry-based application needs to be coupled with the detection of a fluorescent antibody or a fluorescent lipophilic dye to get a robust signal^[62-66]^. Apart from light-scattering-based tools, transmission electron microscopy (TEM) can be used for the quantification of the EVs, but the size of a vesicle can differ largely due to the process of sample fixation, which could lead to swelling or shrinking of the EVs^[67]^.

EVs can also be quantified by measuring the cargo loaded inside the EVs. In this regard, total protein amount, total lipid content, and total RNA have been used as a way of determining EV levels^[57,68]^. Especially the total protein content is still widely used across the research field^[19]^. Although these methods are high throughput and do not require expensive instrumentation, the propensity of measuring non-vesicular contaminants in EV preparation is high. Therefore, measuring the levels of EV-associated protein, for instance, CD63 or CD81, can provide reliable means of quantification but may not reflect the heterogeneity of the EVs^[69-74]^.

Overall, despite the technological advancement and availability of a range of highly sensitive methods, achieving accuracy in EV analytics is still challenging. In addition, the unit particles/mL is an arbitrary unit as one sample measured on different equipment can yield different values. Reflecting on this, we previously performed a meta-analysis of 64 pre-clinical studies using EV as a therapeutic intervention and identified huge variations up to 3 logs in EV doses either based on protein amount or total particles across different studies^[75]^. Moreover, this variation in therapeutic dosing could not be resolved even after segregating the data either based on the type of disease or purification method used or cell source. This clearly reflects that every step from purification to characterization in EV-related research leads to its own variation, which leads to discrepancy in dosing, hence affecting the reproducibility of the work.

## TOOLS FOR IMAGING EVs

**Figure 1:**
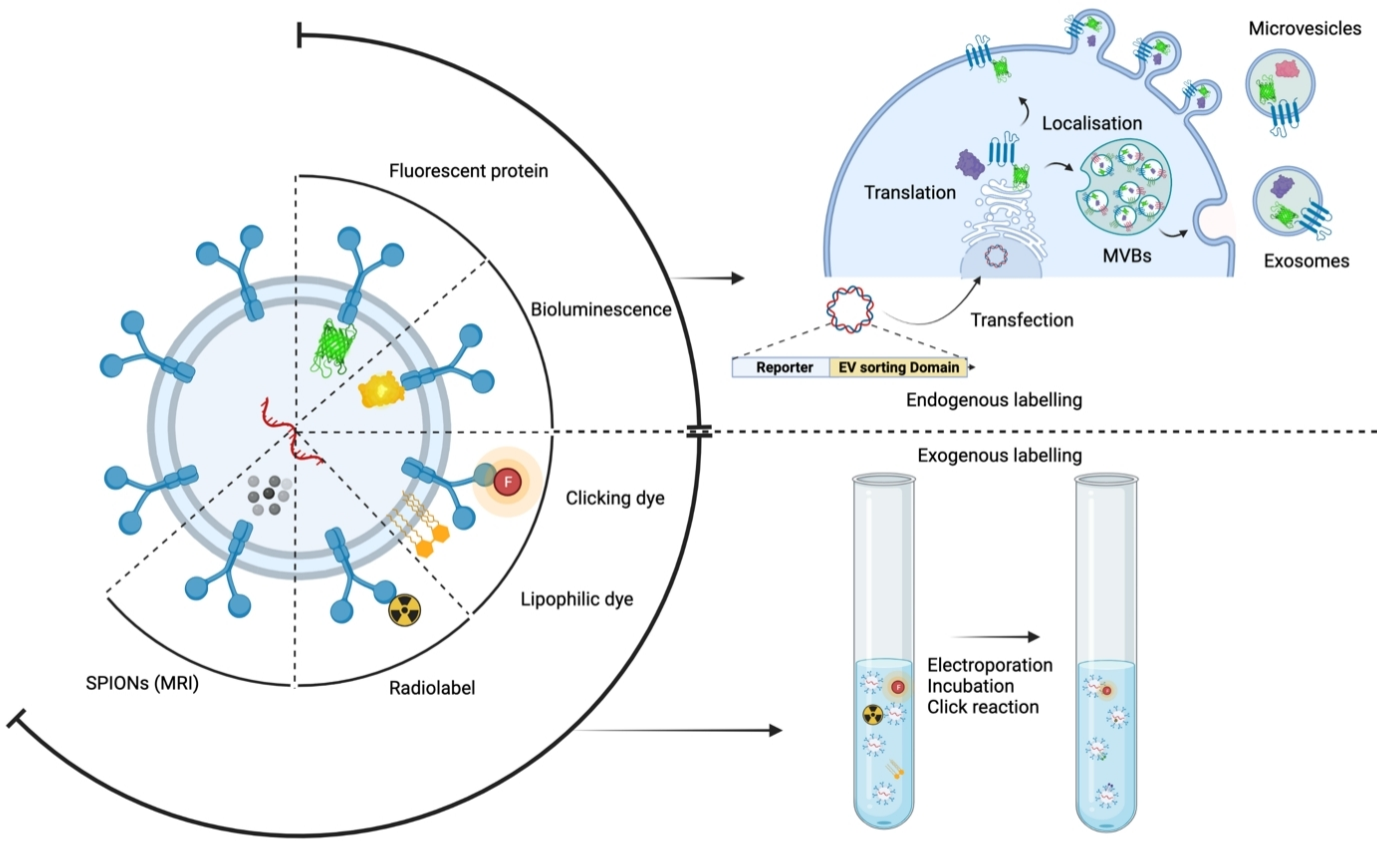
Exogenous and endogenous means of labelling EVs for biomedical applications.

EV labelling can be achieved in two ways, either by general labelling of EV-associated macromolecules or by labelling a specific macromolecule in an EV. There is a range of tools available for labelling EVs with a tracer, both endogenous and exogenous strategies [Figure 1]. This section will discuss a few of the commonly used EV labelling strategies.

### Lipid dyes

Lipophilic tracer dyes have been widely used for EV labelling^[7,76]^. The dyes usually consist of a fluorophore conjugated to a lipophilic functional group which facilitates the insertion of the tracer into the lipid bilayer by non-covalently interacting with EV lipids. Based on this, a number of dyes (for example, PKH67, DiR/DiL/DiD) are available that cover a broad range of the emission wavelengths, including the near-infrared spectrum for better penetration through tissues for *in vivo* applications^[7,76,77]^. Moreover, these dyes allow for quick and efficient labelling of EVs without the need to alter the producer cells. Although being convenient to use and permit labelling of, in theory, all EVs, these dyes tend to aggregate or form micelles and can potentially label non-EV particles^[78]^. Furthermore, there is a considerable risk of transfer of EV-bound dye to the plasma membrane of cells as the interaction is non-covalent. In addition to these limitations, labelling with lipophilic tracer dye has been shown to alter the characteristics of EVs. A similar observation was made by us where labelling of EVs with DiR influenced the biodistribution of EVs *in vivo*^[79,80]^. Apart from lipid anchors, EVs can be labelled with fluorophores by a covalent reaction of fluorophore NHS ester to the amine group of EV surface proteins^[81,82]^. However, these covalent conjugation strategies can potentially alter the EV surface proteome, which may affect their interactions with other proteins. In addition, this approach lacks specificity and may label non-vesicular proteins. Notably, the current generation of dyes and tracers are highly stable and have a half-life of a few days to weeks and do not likely reflect the natural half-life of an EV that is very short^[83,84]^.

### Radiotracers

Apart from fluorescent dyes, EVs can be labelled with a radiotracer (e.g., 99mTc-HMPAO^[85]^, 125I-IBB^[86]^, 111-Indium-oxine^[87]^) either by conjugation to lipophilic groups or to amine groups on the EV surface. MRI has also been used for imaging the biodistribution of EVs. Super magnetic iron oxide nanoparticles can be loaded into EVs either by exogenous loading through electroporation or endogenous loading by feeding cells with 5 nm SPIONs^[88,89]^. The advantage of radiolabelling and MRI is the exceptional sensitivity *in vivo* over other light-based reporters, but due to high infrastructure costs, these technologies are hard to implement in basic science research.

### Fluorescent and bioluminescent proteins

In addition to a variety of exogenous labelling strategies, EVs can be genetically engineered with fluorescent or bioluminescent proteins to label all the EVs or a specific population thereof^[80,82,86,90-92]^. For labelling a specific population, producer cells are genetically engineered to express a reporter protein fused to an EV sorting domain to permit the loading of the reporter protein during EV biogenesis. For example, a fusion protein of CD63 and EGFP can drive the sorting of EGFP into EVs and label 30%-40% of the EV population, each carrying 30-60 EGFP molecules on average^[91]^. This approach is not limited to CD63 since other EV sorting domains can also be exploited for labelling EVs (e.g., CD9, CD81, syntenin, and Gag)^[91,92]^. Although it seems straightforward, the EV engineering efficiency with certain EV sorting domains such as ALIX, SIMPLE and syndecan is relatively low^[91]^. This could be due to the potential loss of the protein’s function due to the fusion of a reporter protein. Overall, genetic engineering approaches provide an efficient way of labelling a specific EV population either with fluorescent proteins (GFP, RFP, *etc*.) or bioluminescent proteins (Gaussia-, Firefly-, and Nano-luciferase). However, these approaches fail to label all EVs and require genetic engineering of the producer cells, which could be challenging for some cell sources. In addition, overexpression of certain EV sorting domains may alter the EV biogenesis pathway or EV proteome, which can influence their biodistribution. An ideal EV reporter or labelling strategy does not exist at this moment. With the multitude of EV labelling strategies available, each has some degree of advantage and disadvantage; therefore, it is essential to select a method based on indication and feasibility.

## *IN VIVO* UPTAKE OF EVs

### The general trend of EVs in vivo biodistribution

Since the first study showing CNS delivery of synthetic siRNA by EVs, interest in EVs as drug delivery technology has gained increasing attention^[93]^. For translational research, it is of uttermost importance to understand the distribution of EVs *in vivo*. Numerous studies in the past decade have showcased EVs *in vivo* biodistribution^[76,80,82,94]^. Since the early use of lipophilic dyes, EV imaging modalities have evolved, and with the development of endogenous labelling strategies, biodistribution can be evaluated with much higher accuracy. The majority of studies have shown that upon systemic administration either by intravenous, intraperitoneal, or subcutaneous injections, EVs tend to primarily accumulate in the liver, lungs, and spleen and to a lesser extent in other organs and tissues such as the brain, muscle, heart, and kidneys^[80,95,96]^. This distribution occurs within minutes of systemic administration of EVs, which is also reflective of the short plasma half-life of EVs. Notably, across literature and based on a meta-study, the peak time of EV accumulation varies drastically from 5 mins to 12 h across studies, but surprisingly the plasma half-life of EVs is always reported short across studies^[96]^. This variability could be due to the differences in EV labelling methods employed in different studies, as different dyes, particularly lipophilic dyes, can shuttle from an EV membrane to a cellular membrane and may not display EV tissue distribution^[7,97,98]^. Secondly, and importantly, the tissue accumulation should be reflective of plasma levels of the EVs, as plasma is primarily the main reservoir of injected exogenous EVs. Therefore, biodistribution models with fast plasma clearance and rapid biodistribution profiles are more logical. In addition, the short half-life of EVs and hepatic tropism is very similar to the majority of synthetic nanoparticles and viral vectors, which indicates that in terms of *in vivo* biodistribution, they all experience similar biological barriers^[99]^. Importantly, this short plasma half-life is observed by various studies utilizing different cell sources of EVs and may imply that cell sources may not play a significant role in regulating EV plasma half-life^[76,80,82,85,94,95]^. The different factors potentially regulating the short plasma half-life of EVs will be discussed later in this review. In contrast, EVs show different half-lives in tissues after being taken up from plasma. These varying levels could be different based on the target cell type in that specific tissue, as phagocytic cells degrade EV-associated cargo much faster as compared to epithelial cells in a given tissue^[80]^. Notably, few studies have been carried out on the *in vivo* biodistribution of EVs at the cellular level. However, the general view is that Kupfer cells in the liver phagocytose the most injected EVs^[87,100-102]^. A similar trend has also been observed in the spleen, where EVs are taken up primarily by splenic macrophages^[102]^. Overall liver and spleen are the major driver of EV uptake *in vivo* and limits the distribution of EVs to other tissues. Notably, the liver and spleen account for one of the multiple barriers EVs experience *in vivo*. Therefore, for achieving targeted delivery to a specific tissue, EVs have to bypass four major biological barriers similar to the majority of other nanoparticle-based drug delivery systems. These four biological barriers are as follows:

#### Endothelium barrier

**Figure 2:**
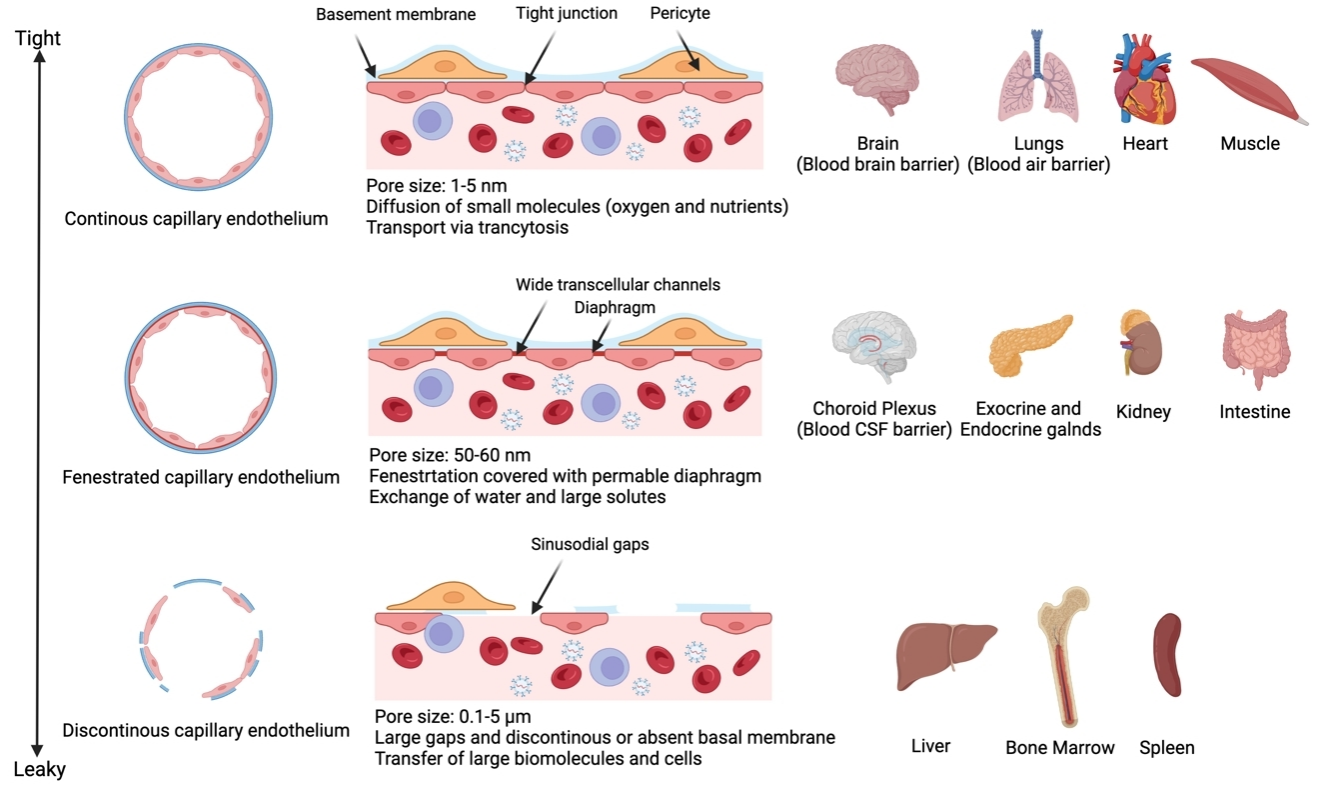
The anatomical structure of different types of endothelium barriers present in different tissues.

One of the initial barriers exogenous EVs encounter upon systemic delivery is the blood-endothelium barrier^[103,104]^. To achieve tissue-specific distribution, EVs need to cross the endothelial monolayer lining the blood vessels of the tissue of interest. The permeability of these endothelial junctions is highly tissue-specific and can have a drastic variation in adsorption efficiencies depending on the type. The endothelium can broadly be divided into three different types based on their permeability, i.e., continuous, fenestrated, and discontinuous^[105]^ [Figure 2]. Continuous endothelium is comprised of tightly connected endothelial cells attached to a continuous basal membrane. The intercellular space between endothelial cells in continuous endothelium is tissue-dependent and can vary from 1-3 nm in healthy conditions and allow diffusion of small molecules such as oxygen and nutrients^[106,107]^. This type of endothelium is mostly found in arteries, veins and capillaries of the brain, skin, lungs, heart and muscle^[107]^.

Fenestrated capillaries, which are present in exocrine and endocrine glands, choroid plexus, gastrointestinal tract, kidney, and renal tubules, are adhered to a permeable continuous basal membrane similar to continuous endothelium but have 50-60 nm intercellular channels for transfer of biomolecules^[106,107]^. These types of capillaries are selective for water exchange and large solutes. The third and the loosest type of endothelium is discontinuous, which has poor basal membrane structure, and the endothelium is loosely arranged and has a variable pore size of 0.1-1 µm pore size^[103,107,108]^. This endothelium predominately forms the sinusoidal endothelial beds in the liver, spleen, and bone marrow^[107]^.

EVs’ *in vivo* biodistribution profile is quite reflective of the endothelial architecture in the body, as organs with discontinuous endothelium, such as the liver and spleen, have the highest accumulation^[80]^, while organs with smaller fenestrated endothelium have a lower accumulation of EVs. Importantly, the pore size of these endothelium varies drastically based on the tissue, and the blood-brain barrier is one of the tightest endothelium^[109]^, therefore transporting of a 100 nm EV across the endothelium is most likely driven by transport across endothelial cells through transcytosis, rather than transport through intercellular junctions^[104,106,110]^. Notably, the transcytosis rate across the blood-brain barrier is relatively low and displays slow kinetics (t1/2 of exocytosis of polymeric nanoparticles: 14.2 h)^[111]^. Since this is an active mechanism, only a limited number of EV particles will be able to induce transcytosis, as the number of interacting receptors on the endothelium is limited^[112]^. This hypothesis is further strengthened by studies showing no significant enrichment in CNS upon administration of EVs *in vivo* into the carotid artery, one of the primary arteries supplying blood to the brain, which should substantially increase the local concentration of EVs near brain endothelium compared to the intravenous route only at the time of administration^[80]^. With such a low efficiency of transcytosis and the relatively short plasma half-life of EVs, achieving biodistribution across the BBB is a challenging task. Therefore, alternative means of achieving CNS distribution could be explored, such as targeting the blood CSF barrier in the choroid plexus, which has larger endothelial fenestrations of 50-60 nm and may circumvent the inefficiency across the blood-brain barrier^[109,113]^. Importantly, the blood CSF barrier permeability is controlled by tight epithelial linings with high vesicular trafficking and may require targeting to induce transcytosis across the epithelium lining. In addition, engineered EVs with targeting domains such as targeting peptides derived from Rabies virus glycoprotein^[93]^ or Folate receptor^[114]^ for enhanced transcytosis across BBB have been employed by multiple studies as a viable strategy for targeted CNS delivery. A similar scenario also exists for other tissues, such as muscle and heart, where endothelium barriers impose a significant challenge towards enhanced biodistribution^[107]^. Importantly, endothelium integrity changes in a diseased state which may lead to a perturbed endothelial barrier that can drive enhanced permeability and retention (EPR) effect. This effect is observed in various pathologies such as cancer, atherosclerosis, and inflammatory diseases, where leakiness of the vasculature drives enhanced nanoparticle uptake and retention into tissue interstitium^[115-117]^. For instance, under neuroinflammation, Yuwan *et al*. showed enhanced EV transcytosis across BBB by interacting with perturbed endothelial cells^[118]^. A similar finding has also been reported in a sepsis model, where the EPR effect enhanced EVs transport across continuous endothelium of the lung^[119]^. Similar findings were also reported recently by Banks *et al*., where LPS-induced inflammation or wheat germ agglutin pre-treatment significantly enhanced EVs *in vivo* biodistribution to the brain and lungs as compared to liver and spleen^[120]^. Although the EPR effect enhances EV uptake in tissues, still the majority of EVs are cleared by the liver and spleen. A similar trend is observed with other nanoparticles, such as lipid nanoparticles, lentiviruses, and polymeric nanoparticles^[99]^.

#### Intravascular barriers

**Figure 3:**
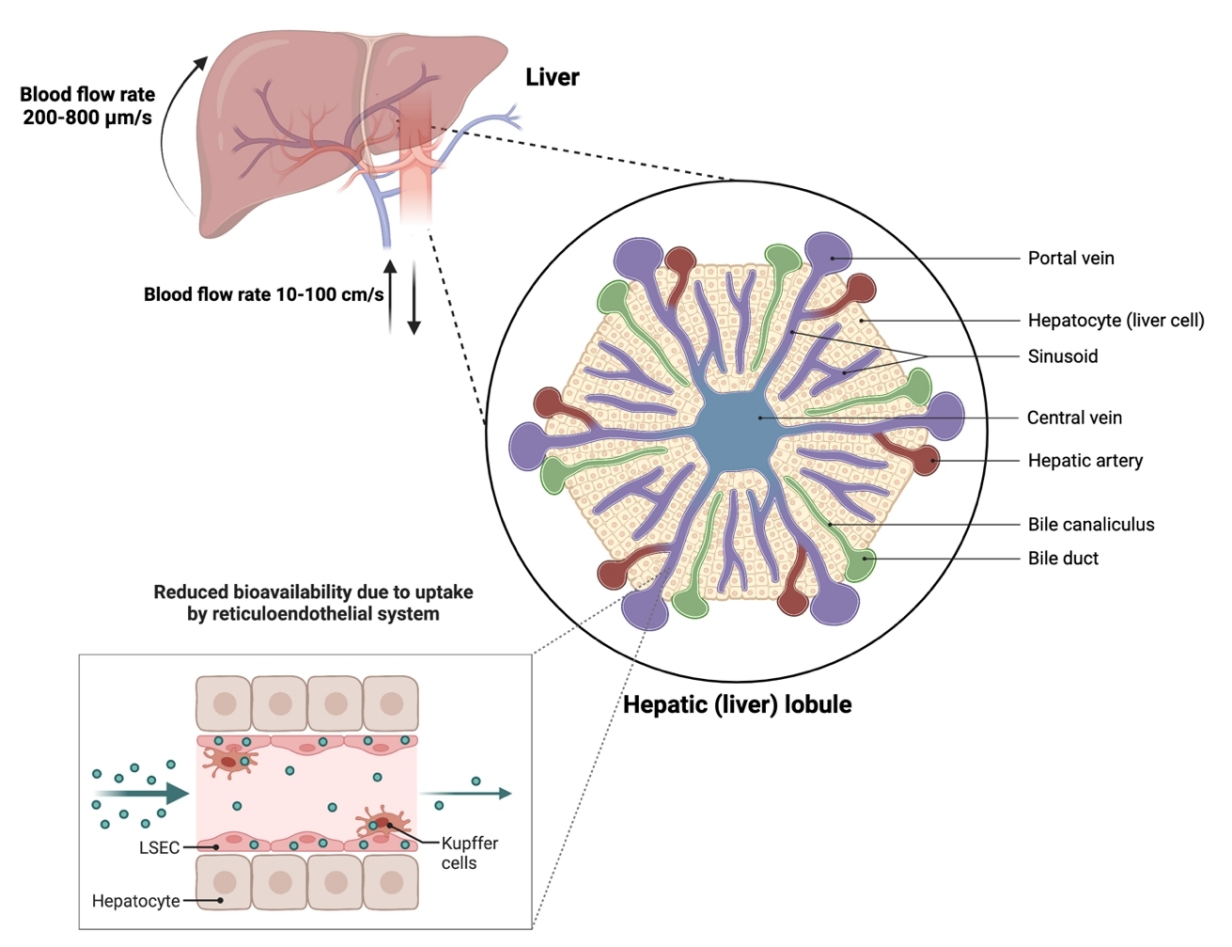
Liver tissue architecture and proposed model of nanoparticle clearance by the mononuclear phagocyte system.

Apart from the endothelium barrier, nanoparticles need to escape the intravascular barriers. The liver and spleen are the major intravascular barriers to nanomedicine, as these organs sequester the majority of the administered biotherapeutic, including EVs, and prevent the distribution to the diseased tissue or tissue of interest. These tissues employ a mononuclear phagocyte system (MPS) to clear the majority of the circulating dose. The MPS is a complex network of immune cells, tissue-resident macrophages and sinusoidal endothelial cells (SECs), which orchestrates with various plasma proteins, such as complement systems, to regulate nanoparticle clearance from the blood^[121]^. In addition, the microarchitecture of the liver and spleen is designed in a way that enhances the interaction of nanoparticles with the MPS system to enhance the clearance^[121-123]^ [Figure 3]. As the nanoparticles move from the peripheral circulation to the liver, the velocity of nanomaterial is reduced from 10-100 cm/sec to 0.2-0.8 cm/sec^[124-126]^. This reduction in blood flow by 100-1,000 folds allows nanoparticles to interact with the MPS system in the liver more efficiently. The blood in the liver enters through portal triads, which are bordered by B and T cells. The decrease in blood flow allows the uptake of the nanoparticles by both B- and T-cells^[122,123]^. The nanoparticle, which escapes the first line of defence, then enters the liver sinusoids. The sinusoids are enriched with SECs and Kupfer cells, a specialized tissue-associated macrophage^[121,123]^. These cells collectively recognize various surface molecules as a trigger for the phagocytosis of nanoparticles. Importantly, this trigger is different for different nanoparticles ranging from size, shape, core properties, surface composition, and charge^[127]^. The residual population of nanoparticles exits through the central vein into the systemic circulation and is ultimately carried back again in the liver or to another MPS system such as the spleen or bone marrow^[122]^. This repetitive process primarily governs the nanoparticle clearance by the liver. The same phenomenon also seems to be responsible for the short half-life and hepatic uptake of EVs. Various studies have shown that the MPS system is primarily responsible for the clearance of EVs, similar to other nanoparticle-based drug delivery vectors, as EVs injected in animals with impaired innate immune- and complement systems have much slower plasma clearance and liver accumulation^[87,119]^. These results are also supported by various studies where either blockade of scavenger receptors on macrophages or the sequestration of exposed phosphatidylserine (PS), one of the primary triggers for macrophage-mediated clearance on the surface of EVs, prevented rapid clearance and liver uptake^[102,128,129]^. Notably, a recent study investigated the protein corona on the surface of EVs in plasma and identified apolipoprotein and complement association with the EVs^[130]^. The EV protein corona had a high overlap with viruses and synthetic nanoparticles^[130,131]^. Importantly complement opsonization of the nanoparticle surface enhances phagocytosis through complement receptor-mediated uptake on macrophages. Interestingly, tumour cell-derived EVs enriched in surface-bound Factor H, one of the negative regulators of complement opsonization, showed lower phagocytic uptake and had higher metastatic potential^[132]^. This clearly implies that EVs are opsonized by complement proteins and are taken up by monocytes and macrophages in the liver (Kupffer cells and/or SECs). Therefore, for enhancing the *in vivo* biodistribution of EVs to extrahepatic tissues, EV engineering strategies to augment MPS clearance are an attractive approach.

#### Extracellular matrix

After penetrating the endothelial barrier, the EV enters the dense and complex environment of the tissue extracellular matrix. Extracellular matrix (ECM) is a non-cellular component of tissue and possesses a mesh-like structure through crosslinking of collagen, fibronectin, elastin, proteoglycans (Hyaluronic acid, decorin, aggrecan, and perlecan, *etc*.) and glycosaminoglycans^[133]^. ECM is composed of gel-like components and provides structural support for the cells in the tissue. Due to the gel-like properties of ECM, the high viscosity limits the fluid flow speed (to 0.1-4 µm/sec)^[134]^. This significantly impairs the Brownian diffusion of the nanoparticles across the ECM^[135]^. In addition, the mesh-like structure of ECM generates steric hindrance for larger nanoparticles such as EVs and partially restricts their diffusion across the tissue^[136]^. Apart from steric hindrance, nanoparticles also experience electrostatic repulsions due to highly polar and negatively charged proteoglycans in ECM^[137]^. These physical forces impose a significant barrier for any nanoparticulate to diffuse into ECM and achieve cellular targeting. Hence, the majority of the delivery by nano particulates is restricted to the periphery of the tissues as the rigidity of ECM inhibits deep tissue penetration^[138]^.

For the transport of EVs in ECM, two different pathways have been described. Active transport involves remodelling and degradation of ECM by cancer-derived EV-associated proteases and glycosidases^[139]^. For instance, EVs are enriched with proteoglycans such as syndecan-1, which can drive the association of heparinase with EVs and further remodel ECM^[140,141]^. Similarly, Membrane type-1 matrix metalloproteinase, sialidase, and insulin-degrading enzymes have been found to be enriched on EVs derived from various cell lines^[142]^. However, there is a lack of substantial evidence on the diffusion of EVs across ECM involving these remodelling enzymes. In contrast, the passive transport of EVs in ECM is much well studied. In an elegant study by Lenzini *et al*., EVs crossed the highly dense ECM by matrix stress relaxation for free diffusion and fast transport with an absence of matrix degradation^[143]^. In addition, Aquaporin 1 present on the EV surface allows for water permeation and deformation to counter steric hindrance in dense networks. Notably, ECM transforms drastically in a diseased state and may affect the diffusion of EVs^[144]^. For instance, tumour ECM is highly dense and imposes a significant barrier for nanoparticle and small molecule movement across the tumour^[145]^. Additionally, inflammation or tissue damage triggers ECM degradation, which may enhance EV diffusion and cellular uptake in tissues^[146]^.

#### Cellular barrier

The last and final barrier for an EV is the cellular barrier. Over the years, much of the focus has been on understanding EV uptake pathways. In an elegant study by Heusermann *et al*., EVs were observed to surf individually on filopodia before being endocytosed, sharing huge similarities with the *in vitro* uptake profile of enveloped viruses^[147]^. Similar to viruses, several energy-dependent pathways such as micropinocytosis, clathrin-dependent, caveolin-dependent or lipid raft-mediated endocytosis processes have been reported to be involved in the uptake of EVs into recipient cells^[148-150]^. Due to the existence of a heterogenous EV population and a variable surface protein composition, the involvement of multiple uptake pathways is expected. The presence of lectins, tetraspanins, integrins and proteoglycans on the EVs surface have shown to be the major driver of EV uptake through energy-dependent endocytosis^[151-155]^. In contrast, evidence of direct fusion with the cell membrane is limited^[156]^.

Importantly, evidence of how the luminal cargo of EVs escapes the endocytic compartment is still largely lacking, with the possibility of either a full fusion between EV and endosomal membrane or complete cargo degradation in lysosomes. This is a critical aspect on which contradictory research exists. Some recent studies have shown that the EVs lack the ability to induce endosomal escape and viral fusogenic proteins are needed for efficient intracellular cargo delivery^[157-159]^. On the contrary, some studies have shown that EVs have an inherent ability to escape from endosomes^[160-163]^. However, there are various factors that could be driving this discrepancy. The difference in cell source could be a potential player as some early developmental cells express ERV ENV genes such as syncytin-1 and syncytin-2, which could potentially induce endosomal escape^[164-167]^. Furthermore, recent evidence suggests that a small percentage of ILVs that are secreted as exosomes tend to retrogradely fuse with the MVB membrane during biogenesis, indicating a possibility that a small proportion of exosomes may be able to induce membrane fusion^[168]^. Importantly, the majority of the studies have addressed biodistribution of the EVs in a specific tissue which may not reflect the bioavailability of the cargo. In addition, with the existence of so many biological barriers, it can be speculated that different cell sources may have similar biodistribution profiles but different bioavailability profiles in different tissues. Therefore, much of the focus should now be on addressing the bioactivity of EVs on a cellular level.

### Strategies to enhance the in vivo pharmacokinetics of EVs

To circumvent the issue of clearance by MPS and enhance extra hepatic delivery of EVs, various engineering strategies, both endogenous and exogenous, have been employed on the surface of EVs.

#### CD47-Don’t eat me signal

CD47 is a cell surface protein expressed on various normal and malignant tissues. Cancer cells utilize CD47 to prevent phagocytosis by interacting with SIRP alpha present on macrophages and thereby evading the innate immune system. In the past decade, various delivery vectors, such as lentiviruses, have been engineered with CD47 to prevent phagocytosis and liver clearance^[169]^. Similar strategies have been explored in the EV space as well, where EVs engineered with CD47 prevented the uptake of EVs in circulating monocytes *in vivo* and extended EV half-life by 3-fold^[170]^. Similar observations were made in other studies where the expression of CD47 prevented macrophage clearance^[171,172]^. Furthermore, the expression of other CD47-like molecules, such as CD55 and CD59, on EVs surface prevented complement activation *in vitro*^[173]^.

#### Albumin

Albumin is the most abundant plasma protein and has a serum half-life of 3 weeks in humans. Albumin interaction with neonatal Fc receptors allows for pH-dependent recycling^[174]^. This recycling property of albumin has attracted a lot of interest in the drug delivery field for enhancing the plasma half-life of a range of biotherapeutics either by directly fusing albumin or introducing albumin binding domains on the surface of the vector for interacting with endogenous albumin^[174]^. Similarly, we recently developed a novel platform for enhancing EV plasma half-life by introducing albumin-binding peptides on the extracellular loop of CD63^[175]^. This EV engineering step allowed for enhanced extrahepatic delivery and enhanced plasma half-life of EVs.

#### PEG

The most commonly used strategy in pharmaceutics to enhance drug pharmacokinetic properties is the conjugation of PEG to prevent hydrophilic and electrostatic interaction of serum proteins with the delivery vector^[176]^. This strategy has been used in various clinically approved delivery vectors, such as LNPs and liposomes^[177]^. Similarly, EVs have also been exogenously engineered with PEG, which in turn enhanced the plasma half-life, decreased hepatic uptake, and improved extrahepatic delivery^[178]^.

Although only a few strategies have been explored in augmenting EVs plasma half-life, it is evident that for enhancing tissue targeting properties of EVs, preventing phagocytic clearance is advantageous.

### Factors governing EVs in vivo biodistribution

#### Route of administration

Route of administration can play a critical role in the *in vivo* biodistribution of EVs, as different injection routes will present a different kind of barrier for EVs to achieve tissue-specific delivery. Even within systemic routes, substantial differences exist between the three most commonly used methods; intravenous (IV), intraperitoneal (IP), and subcutaneous (SC) administration. For instance, drugs administered through SC routes into hypodermis will face adipose tissue and a fibrovascular network of blood and lymphatic vessels^[179,180]^. Drugs smaller than 16 KDa tend to diffuse through blood capillaries, while larger molecular weight drugs such as antibodies or LNPs adopt an indirect route first by diffusion through the cells and ECM, followed by uptake into the lymphatic system to enter the systemic circulation^[179]^. Since lymphatic vessels lack basement membrane, the permeability is much higher as compared to continuous endothelium, and hence allows diffusion of large drug carriers. However, this model is entirely dependent on diffusion through the extracellular matrix, and larger molecules may be rather inefficient in diffusing through the ECM. In addition, lymphocyte-mediated clearance in the lymphatic system will limit the release of cargo into the systemic circulation^[180]^. A similar trend is also observed with EVs, where we and others have seen very small levels of EVs entering systemic circulation^[80]^. Similar barriers also need to be surpassed upon IP injection. The peritoneal cavity consists of a mesothelial cell monolayer attached to multiple layers of connective tissues with embedded vascular and lymphatic system networks^[181]^. In general, two different mechanisms have been proposed for drug uptake from the peritoneal cavity. Similar to SC administration, the nanoparticles enter the systemic circulation through lymphatic drainage. Importantly, IP administration of EVs shows a delay in entering systemic circulation^[80]^, and this could be due to the lymph flow rate, which is 400-700 times slower than the blood flow rate in humans^[182]^.

For local administration routes such as intramuscular, intratumoral, intracerebral, intranasal, or intracerebroventricular, the majority of the EV dose is localized in the injected tissue interstitium, and only a small fraction enters systemic circulation^[76,80,87,183,184]^. This is primarily due to the above-discussed tight endothelial barriers present in these tissues, which regulate the exchange of materials. In addition to the previously discussed strategies to manipulate distribution and pharmacokinetics, several other strategies have been developed for specific tissue targeting, as discussed below.

#### Targeting strategies

The appreciation of EVs as potential therapeutic agents owes not only to their ability to serve as a protective natural delivery vector but also to the numerous reports demonstrating that EVs are targetable. Tissue targeting of functional EVs was successfully demonstrated more than ten years ago, using an approach where the cell source was engineered to produce EVs displaying targeting moieties^[93]^. In this pioneering publication, the authors displayed rabies viral glycoprotein (RVG), which targets the nicotinic acetylcholine receptor, on the EV membrane for functional delivery of encapsulated siRNA to the brain following systemic injections^[76]^. The RVG targeting peptide was enriched on the EV surface by fusion to lysosome-associated membrane protein 2b (Lamp2b), which is known to associate with the EV membrane^[185]^. This parental cell engineering strategy has since then been successfully adapted to display a number of different targeting moieties, including lamp2b fused to T7 peptide^[186]^, αv integrin-specific iRGD peptide^[187]^, tLyp-1^[188]^, and to chondrocyte-affinity peptide (CAP)^[189]^ for EV targeting to the brain, breast cancer, lung cancer, and chondrocytes, respectively. In addition to Lamp2b, targeting peptides have been fused to other EV anchoring proteins, such as different tetraspanins, including CD9 fused to ApoB^[190]^ for brain targeting and CD63 fused to ApoA1^[191]^ for liver cancer (HepG2) targeting. Other examples include the use of the EV-associated lactadherin (C1C2 domain), which has been fused to anti-Her2 single-chain variable fragments to target HER2-positive breast cancer^[192]^, glycosylphosphatidylinositol (GPI) fused to anti-EGFR nanobodies for cancer targeting^[193]^, and platelet-derived growth factor receptors (PDGFRs) fused to the EGFR binding peptide GE11^[194]^. There is thus a great body of evidence showing the successful targeting of EVs by the surface display of a variety of targeting peptides fused to different EV sorting proteins^[195]^. Additionally, overexpression of TNF-related apoptosis-inducing ligand (TRAIL) has been shown to function as a homing mechanism for EVs to lymphoma tumour tissue^[196]^. Another interesting, perhaps somewhat overlooked approach is glycoengineering, which involves the manipulation of the dense glycocalyx of the EVs membrane. In a recent publication, the display of the glycan ligands sialyl Lewis X (sLeX) or Lewis X on EVs were shown to successfully target them to activated endothelial cells and dendritic cells, respectively^[197]^. Albeit the approach of genetic manipulation of the source cell is the most common approach for targeted EVs, there are also other promising EV targeting strategies. One alternative to cell manipulation is to directly modify the purified EVs^[198]^. For instance, surface functionalization has been shown by click chemistry, which has been used to display brain targeting peptides, e.g., c(RGDyK)^[199]^ and RGE^[200]^ that showed increased brain accumulation in a brain ischemic mouse model and in glioma-bearing mice, respectively. Another targeting strategy is to employ aptamers on the EV surface for cancer targeting using cholesterol conjugation. Successful *in vivo* targeting has been shown with EV display of DNA aptamer (AS1411) that binds nucleolin for breast cancer targeting^[201]^, RNA aptamer that binds EGFR, as well as RNA aptamer binding prostate-specific membrane^[202]^. Yet another approach is to functionalize the EV surface by fusion with liposomes. For instance, EV-liposome hybrids employing cRGD decorated liposomes have demonstrated improved tumour targeting^[203]^. Of note, despite the compelling evidence for EVs targetability, the magnitude of targeting is still often unclear. The reports often merely show the fold increase in accumulation of the engineered EVs in the target tissue and do not report the actual percentage of the injected EV dose, which is needed for improved comparison between different methods and for off-target considerations.

## CONCLUSION

Targeted delivery with EVs is still a challenging task. With the existence of various biological barriers, EVs are rapidly cleared by the liver from the plasma, which restricts the distribution to diseased tissue. With the huge overlap of biochemical properties of EVs with other synthetic nanoparticles, understanding and learning from other fields could be applied to refining EV-based therapeutics. Therefore, for achieving superior targeted tissue delivery, multiple EV engineering strategies need to be combined to enhance the plasma half-life of EVs, and engage with endothelial barriers for efficient transcytosis and endosomal escape enhancement for breaching cellular barriers.

## DECLARATIONS

### Copyright

© The Author(s) 2023.
